# Interparticle Spacing Effect among Quantum Dots with High-Pressure Regulation

**DOI:** 10.3390/nano11020325

**Published:** 2021-01-27

**Authors:** Ji-Chao Cheng, Ling-Yun Pan, Xiao-Li Huang, Yan-Ping Huang, Ying-Hui Wang, Shu-Ping Xu, Fang-Fei Li, Zhi-Wei Men, Tian Cui

**Affiliations:** 1 State Key Laboratory of Superhard Materials, College of Physics, Jilin University, Changchun 130012, China; chengjc18@mails.jlu.edu.cn (J.-C.C.); huangxiaoli@jlu.edu.cn (X.-L.H.); huangyp1124@jlu.edu.cn (Y.-P.H.); yinghui_wang@jlu.edu.cn (Y.-H.W.); lifangfei@jlu.edu.cn (F.-F.L.); 2 State Key Laboratory of Supramolecular Structure and Materials, Institute of Theoretical Chemistry, College of Chemistry, Jilin University, Changchun 130012, China; xusp@jlu.edu.cn; 3 School of Physical Science and Technology, Ningbo University, Ningbo 315211, China

**Keywords:** quantum dot, high–pressure, ultrafast dynamic

## Abstract

In this paper, we explore whether interparticle spacing affects steady-state and transient-state optical properties by comparing close-packed CdSe/ZnS–quantum dots (QDs) and CdSe/ZnS–QDs dispersed in polymethyl methacrylate (PMMA). High–pressure is an effective physical means to adjust the interparticle spacing of QDs, which may artificially expand the application of QDs further. The results under high–pressure indicate that it is the reduced interparticle spacing rather than the enhanced quantum confinement effect with volume compression that has a stronger effect on exciton relaxation of CdSe/ZnS–QDs. This work is hoped to help us further understand the effect of interparticle spacing among QDs in various integrated environments.

## 1. Introduction

With the development of preparation methods, the research of nanomaterials has entered the third generation, quantum dot solids (QDSs), in which QDs are close packed with long-range order by various methods, such as lithography, solvent evaporation and surface treatment [1,2,3,4,5,6]. Therefore, the investigations on the interaction among the building units, which are the QDs, are increasing. In a nanoscale, interparticle spacing is a crucial physical factor for interaction among QDs [7], which would influence the interaction among QDs strongly, especially when mono-dispersed QDs (colloidal state) become close-packed QDs (solid state) [8,9,10]. High-pressure is a useful method for exploring the interaction with distance as a parameter. Under pressure, both physical and chemical features of QDs could be modified as a result of compression volume and reducing the interparticle spacing of QDs. Group II–VI QDs are the most extensively researched semiconductor nanomaterials due to their mild preparation methods, high sensitivity to quantum confinement effect, UV–Visible region response and good compatibility for various environments [11,12,13]. Several kinds of phase transition research in CdSe/ZnS–QDs have been reported for both theoretical and experimental aspects [14,15,16]. The Geissler group has calculated that phase transformation pressure from wurtzite to rock salt is 6.0 GPa for CdSe–QDs [15]. However, this pressure could be elevated by ZnS capping, due to a lattice mismatch between CdSe and ZnS [17]. Because of large differences in elasticity modulus, the phase transition pressure was observed at ~7.0 GPa inside the CdSe core without collapse of the ZnS shell [14,15,16]. Various reports indicate that QDs experienced anisotropic compression and an energy gap increase before their phase transition [17,18].

The compression of QDs can strongly affect the exciton dynamics, such as exciton recombination, carrier hopping and charge transport [19,20,21,22]. Carrier mobility could be improve by seven times when the pressure increased from 0.1 to 5 MPa in PbS–QDSs [23], while a faster relaxation process was observed in mono-dispersed CdTe solution when the pressure was elevated to 6.8 GPa [24]. The Jin group reported electron transfer and an Auger recombination rate of CdSe/ZnS–QDs–anthraquinone in cyclohexane with a pressure of up to 2.4 GPa. The electron transfer process was considered to be enhanced and the Auger process was suppressed in CdSe/ZnS–QDs–anthraquinone [25].

Here, the volume and interparticle spacing effect are distinguished by compressing isolated and close-packed CdSe/ZnS–QDs. The results indicate that interparticle spacing has a greater effect than the volume on the exciton dynamics when the distance between QDs becomes closer. The surface trap state of QDs assists the exciton delocalization process among QDs.

## 2. Materials and Methods

High–pressure was generated by a diamond anvil cell (DAC). UV–visible absorption spectra were completed by a home-built in situ high-pressure absorption system. The incident white light (Ocean, New York, USA, HL-2000, 360–2400 nm) was focused into the DAC and transmitted light was collected by a spectrometer (Avantes, Apeldoorn, Nederland, AvaSpec-2048 × 16, 200–1160 nm). The in situ high-pressure photoluminescence (PL) spectra were achieved by the LabRAM HR Evolution system (Palaiseau, France) with 325 nm excitation. The in situ high-pressure ultrafast dynamic was tested by a home-built transient absorption system. The incident laser beam (800 nm, 35 fs, Spitfire, Spectra-Physics, Santa Clara, CA, USA) was split by a beam splitter (7:3). The 70% laser was used to produce a 400 nm pump pulse through a β-BaB_2_O_4_ (BBO) crystal as a pump beam. The 30% laser passed through a cuvette full of H_2_O to generate a supercontinuum as a probe beam. PL lifetime under high–pressure was obtained by a time-correlated single-photon counting (TCSPC) coupled DAC, which was described in [26].

For the preparation of close-packed QDs (named CdSe/ZnS–QDs), pristine CdSe/ZnS–QD (Mesolight, Suzhou, China) solution was cast on a slide and transferred into the DAC. For the preparation of isolated QDs (named CdSe/ZnS–QDs–PMMA (polymethyl methacrylate)), CdSe/ZnS QD solution was blended with PMMA solution with a 1:1 volume ratio and dried on a glass slide. Then, the dried mixture was transferred into the DAC. The morphology of QDs was characterized by high-resolution transmission electron microscopy (HR-TEM, JEM-2200FS JEOL, Tokio, Japan). All experiments were carried out at room temperature.

## 3. Results and Discussion

**Figure 1:**
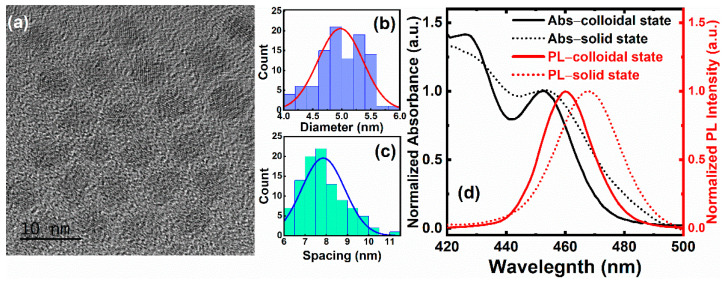
(**a**) High-resolution transmission electron microscopy (HR-TEM) of CdSe/ZnS–quantum dots (QDs). (**b**) Chart for particle size distribution. (**c**) Interparticle spacing (center to center) distribution of CdSe/ZnS–QDs by HR-TEM. (**d**) UV–Vis absorption and photoluminescence (PL) spectra of CdSe/ZnS–QDs in the colloidal state (toluene) and solid state (film).

Figure 1a shows the morphology of pristine QDs and the mean diameter is ~5 nm. Figure 1c shows that the interparticle spacing (center to center) remains ~8 nm in ambient conditions. It also shows an inhomogeneous distribution. Figure 1d illustrates steady-state UV–Vis absorption and PL spectra of CdSe/ZnS–QDs in the colloidal and solid state in ambient conditions. Two absorption peaks, ~452 nm and 427 nm, are shown in Figure 1d, which correspond to 1S(e)-1S_3/2_(h) and 1S(e)-2S_3/2_(h), respectively [27]. For PL spectra, the band-edge emission of CdSe/ZnS–QDs in both states is located at ~460 nm with a ~18 nm full width at half maximum (FWHM) Gaussian shape.

**Figure 2:**
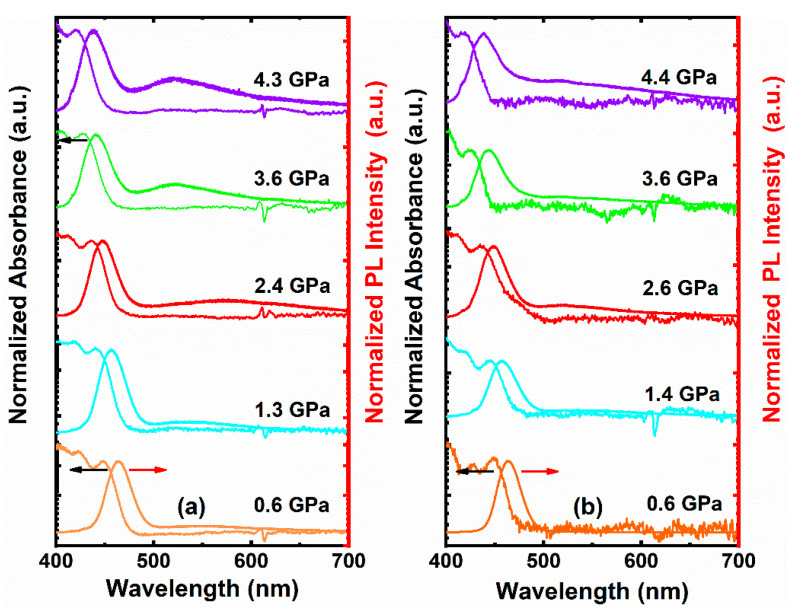
Pressure-dependent UV–Vis absorption and PL spectra of (**a**) CdSe/ZnS–QDs and (**b**) CdSe/ZnS–QDs–polymethyl methacrylate (PMMA).

**Figure 3:**
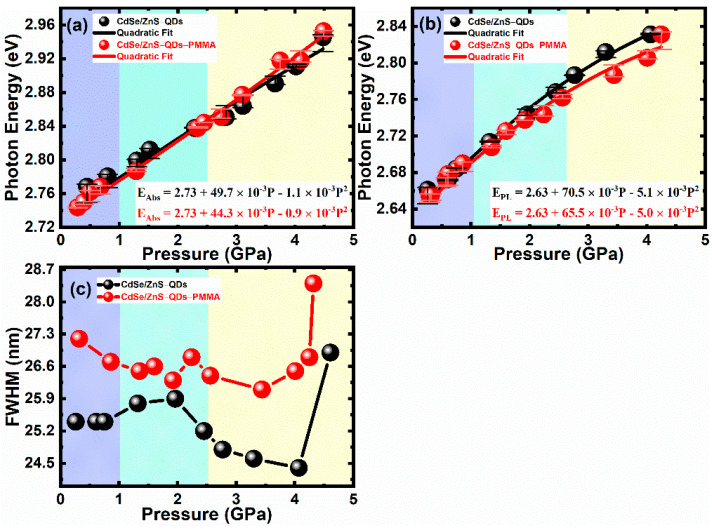
Quadratic fit by the equation in pressure-dependent (**a**) UV–Vis absorption, (**b**) band-edge PL spectra of CdSe/ZnS–QDs and CdSe/ZnS–QDs–PMMA. (**c**) The relation between pressure and FWHM of CdSe/ZnS–QDs and CdSe/ZnS–QDs–PMMA, according to band-edge PL spectra under pressure.

Figure 2a,b illustrate the pressure-dependent UV–Vis absorption spectra of CdSe/ZnS–QDs and CdSe/ZnS–QDs–PMMA. The peak shifts to higher energy with increasing pressure as result of an enhanced quantum confinement effect [28,29,30,31], which originates from the compressed QDs’ volume. The pressure-dependent band-gap changes can be described by E = E_0_ + αP + βP^2^ [28,29], where E is energy in eV; P is pressure in GPa; α is relative bulk modulus; β is the derivative of pressure. The fitting value in Figure 3a,b is close to the reported results [29]. The tendency of shift shows a slight difference between the two samples. The difference begins at 2.8 GPa. By considering the Young’s modulus of PMMA (2.7–3.2 GPa), this phenomenon could be due to compression on isolated QDs in the PMMA matrix. On the contrary, QDs are close packed in CdSe/ZnS–QDs and the elastic modulus of QDs increased with volume compression. As shown in Figure 2, the different tendency of shift is more obvious in PL spectra. Firstly, the band-edge change in CdSe/ZnS–QDs–PMMA is less than that in CdSe/ZnS–QDs with pressure >1.0 GPa, which is shown in Figure 2a,b. Secondly, two emission peaks were generated in both samples with pressure >1.0 GPa and were enhanced. They are band-edge emissions within 435–465 nm for both systems and a long-wavelength emission within 500–600 nm for CdSe/ZnS–QDs, 500–650 nm for CdSe/ZnS–QDs–PMMA. Since phase mismatching induces trap states that will quench instead of enhance emission, long-wavelength emissions may be from surface trap states [32,33]. In CdSe/ZnS–QDs, the trap-state emission is rather weak as compared with a band-edge emission at less than 2.5 GPa. The peak shifts from 555 nm at 0.6 GPa to 547 nm at 1.3 GPa, and then shifts to 567 nm at 2.4 GPa, while the spectra begin to show a Gaussian shape with pressure up to 2.5 GPa. A 520 nm peak shows no shift with a pressure increase. The irregular changes of spectra at less than 2.5 GPa are due to the trap states among particles, which are formed when the particles are not packed closely. With the pressure increasing, particles are closer and the surface trap-state emission shows a stronger effect, while in CdSe/ZnS–QDs–PMMA, the trap-state emission is rather flat with a slight 520 nm peak. This is the result of surface modification by PMMA, which reduces the quantity of the surface trap state [34,35].

The amplitude of the surface trap-state emission in CdSe/ZnS–QDs is larger than that in CdSe/ZnS–QDs–PMMA under the same pressure. This phenomenon can be explained by the interparticle spacing of QDs [36]. With a pressure increase, spacing among QDs without PMMA is effectively reduced, which increases the possibility of electron hopping among QDs [37]. As shown in Figure 3c, a constant FWHM (25.3 ± 0.9 nm for CdSe/ZnS–QDs, 26.6 ± 1.5 nm for CdSe/ZnS–QDs–PMMA) of the band-edge emission and broadened FWHM of the surface trap-state emission confirm the enhancement of electron delocalization in these states with a pressure increase. Meanwhile, the position of surface trap-state emission peaks is not changed with pressure up to 2.5 GPa, which is located at 520 nm for both samples. Thus, the trapping rate remains constant and electron delocalization may be enhanced due to the reduced interparticle spacing. In this case, steady-state spectra show a time-integrated effect, in which more electrons relax into the surface trap-state because of the faster dumping rate of these states. Both the surface trap-state emission and electron hopping will be retarded in CdSe/ZnS–QDs–PMMA because of PMMA. Briefly, a shift of the emission peak in both systems is induced by enhancing the quantum confinement effect with volume compression. The behavior of the surface trap-state emission strongly depends on the interparticle spacing of QDs. The interparticle spacing effect can also be observed in the transient state.

**Figure 4:**
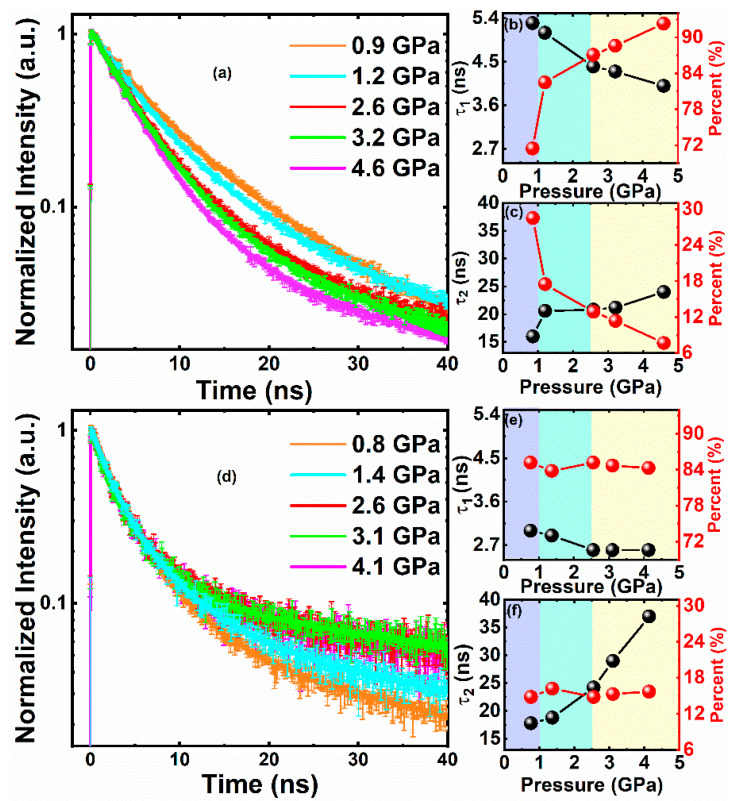
(**a**,**d**) Normalized pressure-dependent PL lifetime of CdSe/ZnS–QDs and CdSe/ZnS–QDs–PMMA, respectively. (**b**,**e**) Functional relationship between pressure and τ_1_ (percent) of CdSe/ZnS–QDs and CdSe/ZnS–QDs–PMMA, respectively. (**c**,**f**) Functional relationship between pressure and τ_2_ (percent) of CdSe/ZnS–QDs and CdSe/ZnS–QDs–PMMA, respectively.

For close-packed QDs, pressure affects band-edge decay early, which is shown in Figure 4. The length of rising time is shorter than decay time by about 100 times. Figure 4b shows an accelerated fast component of τ_1_ (5.3 ns@0.9 GPa to 4.0 ns@4.6 GPa) as well as its increased amplitude (72%@0.9 GPa to 92%@4.6 GPa), which are close to previous reports [38]. Since the fast component, τ_1_, corresponds to the surface trap-state lifetime, this is consistent with the steady-state result. The rate of electron transfer is accelerated by reduced interparticle spacing among QDs, which dumps the surface trap state and accelerates the trapping process with a pressure increase. Meanwhile, pressure affects the longer time of CdSe/ZnS–QDs–PMMA in Figure 4f, which shows a retarded slow component, τ_2_ (17.8 ns@0.8 GPa to 37.0 ns@4.1 GPa), as well as its unchanged amplitude (15%@0.8 GPa to 16%@4.1 GPa). Since τ_2_ corresponds to the intrinsic recombination process, this could be due to the transition from direct band-gap to indirect band-gap. The recombination slows down with a pressure increase because of less alignment of the conduction band and valence band. On the other side, τ_1_ in Figure 4e remains almost constant (3.0 ns@0.8 GPa to 2.6 ns@4.1 GPa) with pressure up to 4.1 GPa, as well as its amplitude (85%@0.8 GPa to 84%@4.1 GPa), which indicates the absence of QD interaction. These results suggest that QD interaction is through the surface trap state, which is accelerated with reduced interparticle spacing. Furthermore, the interparticle spacing effect on a deep timescale was investigated.

**Figure 5:**
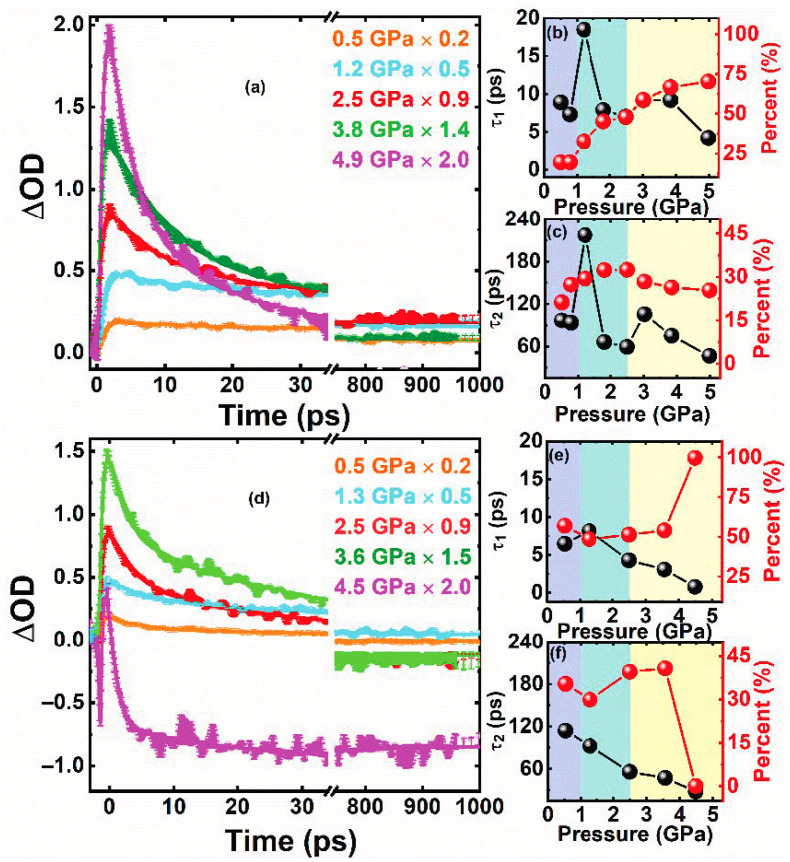
(**a**,**d**) Normalized ultrafast carrier dynamics of CdSe/ZnS–QDs and CdSe/ZnS–QDs–PMMA, respectively. (**b**,**e**) Functional relationship between pressure and τ_1_ (percent) of CdSe/ZnS–QDs and CdSe/ZnS–QDs–PMMA, respectively. (**c**,**f**) Functional relationship between pressure and τ_2_ (percent) of CdSe/ZnS–QDs and CdSe/ZnS–QDs–PMMA, respectively.

As indicated in Figure 5a,d, the relaxation process becomes fast in both systems under pressure up to the limitation of the dynamics signal (4.5 GPa). ∆OD represents the change of absorbance between pumped and unpumped samples. Furthermore, CdSe/ZnS–QDs show a slower relaxation than CdSe/ZnS–QDs–PMMA under pressure. PMMA passivates the surface trap states of QDs, which has been explained by steady-state spectra and PL lifetime. The dynamic behavior of CdSe/ZnS–QDs–PMMA is the intrinsic behavior of excited state relaxation in QDs and accelerates rapidly with a pressure increase, as shown in Figure 5e,f. This is due to the reduction of QD size, which enhances the Coulomb interactions between electrons and holes, and makes the annihilation rate increase rapidly. The negative signal appears with pressure above 4.0 GPa. Biexciton is considered to be generated with increased QD density with pressure increases or volume reductions. As proved by Klimov and Bawendi et al., the biexciton band will be formed below the single exciton band (∆ = 14 meV with 3.5 nm size) and thus an induced absorption is formed transiently [39,40]. Furthermore, the electron–electron and hole-hole interactions may overwhelm the exciton–exciton attraction with a reduction of the confinement effect, which also reduces the shift between the single exciton band and biexciton band. Thus, the amplitude of induced absorption is larger in CdSe/ZnS–QDs–PMMA than that in CdSe/ZnS–QDs because of less efficient delocalization of the biexciton for the isolated QDs in the PMMA matrix. However, the dynamic behavior of QDs is complicated by the interaction among particles. As a whole, the interaction among particles enhances the relaxation of electrons to the ground state. With pressure <2.4 GPa, as indicated in Figure 5b,e, the fast component of τ_1_ in CdSe/ZnS–QDs (59.4 ps) is slower than that in CdSe/ZnS–QDs–PMMA (55.8 ps). As analyzed in the result of the pressure-dependent PL lifetime, the surface trap-state emission occupies a large proportion of CdSe/ZnS–QDs. Therefore, we consider that the delocalization of excited electrons, which is caused by the interaction among adjacent QDs, slows down the excited state relaxation with pressure <2.0 GPa. The relaxation time in CdSe/ZnS–QDs is faster than that in CdSe/ZnS–QDs–PMMA with a pressure of 1.0~2.0 GPa. This may be due to competition between the Coulomb effects inside each QD and the interaction among QDs. In this pressure range, the effect of the reduced interparticle spacing is smaller than that of the enhanced Coulomb interaction, which is caused by compression volume. The compressibility of QDs is 1.4 × 10^−2^ GPa^−1^ with 2.0 GPa, which is close to previous reports [41]. However, this acceleration in CdSe/ZnS–QDs is much less obvious than in CdSe/ZnS–QDs–PMMA. In other words, electron delocalization does not change significantly with pressure less than 1.0 GPa. In summary, the pressure-dependent relaxation in QDs is a comprehensive process, which includes volume compression accelerating intrinsic relaxation inside each QD and reduced interparticle spacing slowing down relaxation by carrier delocalization. According to the experimental results, enhanced electron delocalization caused by reduced interparticle spacing cannot be ignored within the entire pressure range (below the phase transition). On the other side, the delocalization process is not sensitive to pressure. Thus, the influence of the interparticle spacing on the electron relaxation must be considered when the spacing among particles is close enough. These results are supposed to be significant for studying the influence of the interface strain inside QD devices on the PL and photoelectric effect.

## 4. Conclusions

In this work, both steady-state and transient-state spectra of isolated and close-packed CdSe/ZnS–QDs under pressure are detected. In the steady-state absorption spectra, volume compression-correlated behaviors show no obvious differences between the two samples. Meanwhile, in the steady-state PL spectra, the effect of the surface trap state on QDs shows stronger emissions in isolated QDs than that in close-packed QDs with a pressure increase. Combined with the TCSPC results, this suggests that QD interaction is through the surface trap state, which is accelerated with reduced interparticle spacing. Further dynamics analysis on the picosecond level shows that interparticle spacing influences the delocalization of electrons when the spacing among particles is close enough. We hope our results not only provide optical characters of QDs under pressure but make contributions to fundamental explorations of QDs in various compatible, strain contained and integrated systems, such as QD solid-based devices.

## Data Availability

The data presented in this study are available on request from the corresponding authors.
